# Pituitary metastasis of salivary gland carcinoma mimicking hypophysitis: A case report and literature review

**DOI:** 10.1016/j.ijscr.2023.108522

**Published:** 2023-07-23

**Authors:** Takeyoshi Tsutsui, Kosuke Hayashi, Masashi Oda, Shinpei Kada, Naohiro Yamazoe, Masaaki Saiki

**Affiliations:** a Department of Neurosurgery, Japanese Red Cross Otsu Hospital, Shiga, Japan; b Department of Otolaryngology, Japanese Red Cross Otsu Hospital, Shiga, Japan

**Keywords:** Pituitary metastasis, Salivary gland cancer, Hypophysitis

## Abstract

**Introduction:**

Pituitary metastases from salivary gland carcinomas are rare. Moreover, pituitary metastasis and hypophysitis exhibit neuroimaging similarities that complicate the diagnosis in patients receiving immune checkpoint drugs.

**Presentation of case:**

We present a case of pituitary metastasis derived from a sublingual gland carcinoma; this case posed a challenge in the differential diagnosis of hypophysitis. A 52-year-old male patient presented with anorexia and visual disturbances. The patient was previously diagnosed with sublingual gland carcinoma that necessitated surgical intervention consisting of tumor resection and residual lymph node dissection. Subsequently, the patient underwent immune checkpoint blockade therapy following platinum-based chemotherapy. Magnetic resonance imaging revealed the presence of an intrasellar tumor infiltrating the dura mater, cavernous sinus, and pituitary stalk with isointensity on T1 and T2 weighted images and homogeneous gadolinium enhancement. Despite the initial suspicion of hypophysitis, diagnostic treatment with systemic corticosteroids failed to induce significant tumor reduction. Diagnostic clarification was achieved via an endoscopic transsphenoidal biopsy, which confirmed the histological diagnosis of pituitary metastasis from the prior sublingual gland adenocarcinoma. Radiotherapy was administered as a therapeutic intervention.

**Discussion:**

The case report highlighted the rarity of metastases from salivary gland carcinoma to the pituitary gland and emphasized the challenges in distinguishing between pituitary metastasis and hypophysitis based on imaging studies alone, particularly in patients receiving immune checkpoint inhibitors.

**Conclusion:**

Given the rarity of this condition and its neuroimaging similarities with hypophysitis, pathological confirmation is imperative for a definitive diagnosis.

## Highlights

• We present a case of pituitary metastasis derived from a sublingual gland carcinoma.
• Under the administration of immune checkpoint drugs, this was a rare condition having neuroimaging similarities with hypophysitis.
• Diagnostic clarification was achieved via an endoscopic transsphenoidal biopsy.
• Pathological confirmation is imperative for a definitive diagnosis in such cases.

## 1 Introduction

The pituitary gland is an uncommon metastasis site, constituting only 0.4 % of all intracranial metastatic tumors; moreover, less than 1 % of pituitary tumors require surgical intervention [1]. Most pituitary metastases are attributable to lung and breast cancers, with prostate and kidney cancers contributing to a lesser extent [2]. The incidence of pituitary metastases from salivary gland adenocarcinomas is also low.

In this report, we describe a case of pituitary metastasis from a sublingual adenocarcinoma during treatment with immune checkpoint inhibitors. The differentiation of pituitary metastases from hypophysitis is complicated in patients receiving immune checkpoint inhibitors [3] because hypophysitis is a well-known adverse effect in these patients [4]. Hypophysitis is characterized by diffuse enlargement of the pituitary gland and stalk, as well as homogenous contrast enhancement on magnetic resonance imaging (MRI) [5]. In the present case, this neuroimaging characteristic and the use of immune checkpoint inhibitors made differentiation from autoimmune hypophysitis challenging compared to previous reports [[6], [7], [8], [9], [10]]. This case report has been reported in line with the SCARE criteria [11].

## 2 Presentation of case

**Fig. 1:**
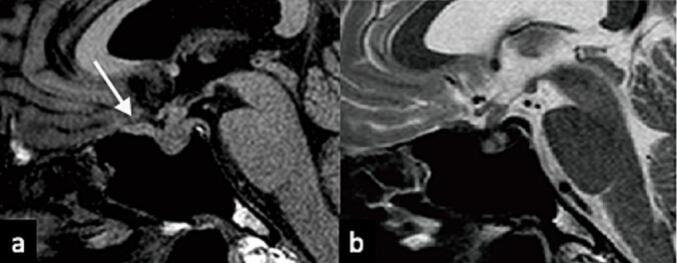
Initial image before treatment showing an intrasellar tumor with an extension to the dura mater (white arrow), cavernous sinus, and pituitary stalk. Sagittal T1-weighted image without contrast showing the isointense tumor without high signal in the posterior lobe (a). Sagittal T2-weighted image showing the isointense tumor (b).

**Fig. 2:**
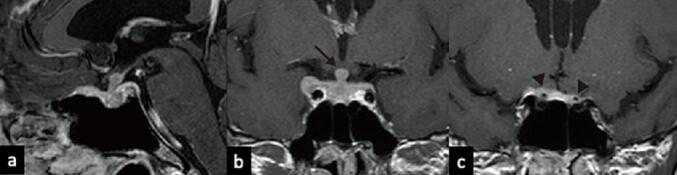
T1-weighted image with contrast before treatment demonstrates the intrasellar tumor without the optic chiasm compression (arrow) (a: sagittal, b: coronal). Coronal T1-weighted image with gadolinium contrast showing optic nerve compression (arrowhead) by the tumoral invasion of the dura (c).

A Japanese man, aged 52, presented at our hospital with anorexia that had persisted for several months. The patient, a non-smoker with no notable family medical history, received sertraline hydrochloride as an antidepressant. His medical history included treatment of sublingual adenocarcinoma with resection and radiation therapy six years prior and supraclavicular lymph node metastasis with resection and radiation therapy five years prior. After platinum-based chemotherapy for lung and bone metastases, the patient also received nivolumab, a programmed death 1 (PD-1)-targeted immune checkpoint inhibitor. During the initial examination, haematological testing revealed hypopituitarism, including secondary adrenocortical insufficiency and hypothyroidism, but not diabetes insipidus. The patient's immunoglobulin G4 serum level was 52.0 mg/dL, and other biochemical examination results were as follows: angiotensin-converting enzyme, 13.6 U/L; soluble interleukin-2 receptor, 684 U/mL; and T-SPOT, negative. MRI showed swelling in the pituitary gland and stalk with isointensity on T1 and T2 weighted images [Fig. 1a and b] and homogeneous gadolinium enhancement [Fig. 2a - c]. Pituitary gland swelling extended into the pituitary stalk, cavernous sinus, and the dura mater of the planum sphenoidale without compressing the optic chiasm [Fig. 2b]. No other lesions were found in the cranium except around the sella turcica. Thus, we suspected autoimmune hypophysitis caused by immune checkpoint inhibitors, and steroids and thyroid hormone supplements were administered.

**Fig. 3:**
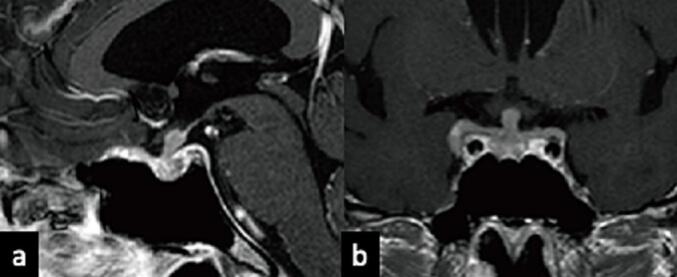
Preoperative T1-weighted image with contrast after steroid administration showing no tumor shrinkage (a: sagittal, b: coronal).

**Fig. 4:**
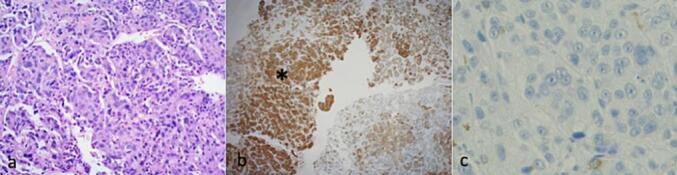
Hematoxylin and eosin (original magnification ×100) staining of resected specimens showing solid atypical cells with clear nucleoli or high nuclear-cytoplasmic ratio infiltrated in the glandular arrangement (a). Immunohistochemical analysis showing the invasion of pankeratin-positive carcinoma tissue (asterisk) into the glandular structures of the anterior pituitary gland (original magnification ×20) (b). p53 positivity (original magnification × 400) (c).

**Fig. 5:**
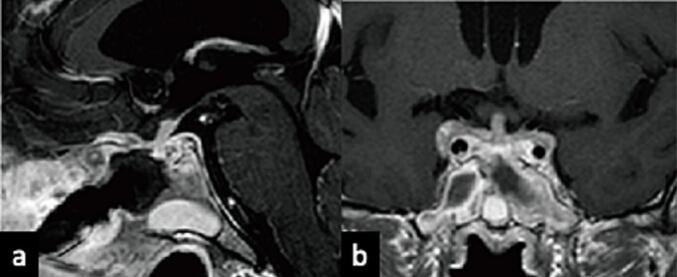
T1-weighted image with contrast after the radiation therapy showing tumor reduction (a: sagittal, b: coronal).

One month later, the patient returned with visual impairment; their visual acuity was normal in the left eye but 20/200 in the right eye, attributed to compression caused by invasion of the optic canal [Fig. 2c]. Thus, pulse corticosteroid therapy was administered as a diagnostic treatment. However, there was no clear shrinkage of the pituitary tumor on MRI [Fig. 3a and b]; thus, a tissue biopsy was performed following a new oculomotor nerve palsy in the right eye. Histological examination revealed cancerous tissue invasion of the anterior pituitary gland [Fig. 4a]. Immunohistochemical staining was positive for pankeratin and p53 [Fig. 4b and c] but negative for synaptophysin, chromogranin A, p40, and human epidermal growth factor receptor-2. Based on these findings, we confirmed pituitary metastasis from the previous salivary gland adenocarcinoma. After intensity-modulated radiotherapy to suppress symptom progression and control the tumor, visual function improved in both eyes; the right eye improved to the photic valve, and the left eye improved to the exponential valve. The patient was discharged several days after radiation therapy and continued to receive steroid supplementation. Post-radiotherapy MRI showed tumor regression [Fig. 5 a and b]. The patient was satisfied with the treatment he received. Imaging was performed every few months thereafter.

## 3 Discussion

In 2020, Sam et al. published a systematic review of pituitary metastases, presenting a comprehensive analysis of 657 pituitary metastases cases [1]. The primary symptoms included dysfunction of the anterior lobe, diabetes insipidus, visual field defects, diplopia, visual impairments, and headaches. In one-third of the cases, the diagnosis was concurrent with at least one additional brain metastasis. The study found that while the extent of resection did not significantly impact survival, overall survival was notably prolonged in patients who underwent radiotherapy.

To our knowledge, only five cases of hematogenous metastases from salivary gland carcinoma to the pituitary gland have been reported [Table 1] [[6], [7], [8], [9], [10]], emphasizing their rarity. In this case report, the high frequency of primary parotid gland cancers may reflect the elevated prevalence of parotid gland tumors among salivary gland tumors. The presenting symptoms in the current case, including hypopituitarism and visual impairment, were consistent with those of other pituitary metastases. All prior cases were accompanied by optic chiasm compression and suprasellar invasion. Radiation therapy was frequently used as an adjuvant treatment.

**Table 1:** Summary of cases of pituitary metastasis from salivary gland cancer.

Author	Age	Sex	Origin	Time from primary cancer	Main symptom	Histological classification	Postoperative treatment	Overall survival
McCutcheon et al., 2001	47	Male	Parotid	18 m	Hypopituitarism, DI	Salivary ductal adenocarcinoma	WBRT(30Gy)	7 m
Kawamata et al., 2006	78	Female	Parotid	4y	Hyponaturemia	Adenoid cystic carcinoma	gamma knife	N.D.
Woo et al., 2015	81	Female	Parotid	10y	Visual impairment	Acinic cell carcinoma	none	1 m
Hughes et al., 2016	72	Female	Parotid	26y	Visual impairment	Adenoid cystic carcinoma	RT(37.5Gy)	N.D.
Zaky et al., 2021	61	Female	Buccal mucosa	3y	Diplopia, headaches, DI	Adenocarcinoma	not mentioned	N.D.
Present case	53	Male	Sublingual	8y	Hypopituitarism, visual impairment	Adenocarcinoma, not otherwise specified	RT(60Gy)	survive

N.D. = not described DI = Diabetes insipidus WBRT = whole brain radiation therapy RT = radiation therapy.

In contrast to prior case reports of pituitary metastases from salivary gland carcinoma, immune checkpoint inhibitor use was a noteworthy factor for the hypophysitis misdiagnosis in the present case. Immune checkpoint inhibitors were not administered in previous reports. Although the efficacy of chemoradiotherapy for treating metastatic salivary gland cancer is limited [7], previous studies have demonstrated the efficacy of nivolumab for relapsed or metastatic salivary gland cancer [12]. However, the distinction between pituitary metastasis and hypophysitis was further complicated by the use of immune checkpoint inhibitors.

Hypophysitis, often associated with immune checkpoint inhibitors [13], is characterized by MRI findings of an enlarged triangular or dumbbell-shaped gland with a thickened and non-deviated stalk [14]. This neuroimaging characteristic was reminiscent of the MRI findings in the present case. In this case, the pituitary gland and stalk were enlarged with homogeneous contrast enhancement, a specific finding in contrast to pituitary metastasis, indicated by a large lesion with heterogeneous contrast enhancement on MRI [3]. The absence of other intracranial metastases and small suprasellar lesions supported the diagnosis of hypophysitis.

This case report emphasizes that pituitary metastasis and hypophysitis exhibit neuroimaging similarities that may complicate the diagnosis, particularly in patients undergoing immune checkpoint treatment for salivary gland cancer. However, timely diagnosis is critical in preventing the progression of symptoms, and, owing to the rarity and similarities of the conditions, a pathological examination is necessary for a definitive diagnosis of pituitary abnormalities. In this case, secondary hypothyroidism was atypical for anti-PD-1-induced hypophysitis [4], while the tumoral extension into the cavernous sinus and the dura mater suggested pituitary metastasis rather than hypophysitis [3]. Therefore, considering these findings, a tissue biopsy could have been performed before pulse corticosteroid therapy as a diagnostic treatment.

Finally, we recommend transsphenoidal surgery for all pituitary metastases from salivary gland carcinoma cases with, in most cases, radiotherapy as the postoperative treatment. The efficacy of radiotherapy for pituitary metastases has been reported [1], and a well-defined adjuvant therapy plan also requires surgical pathology.

## 4 Conclusion

Here, we report a case of pituitary metastasis from salivary gland carcinoma, making the differential diagnosis of hypophysitis challenging. Pathological diagnoses are important in pituitary metastasis, and radiation therapy often follows the postoperative treatment for pituitary metastasis.

## CRediT authorship contribution statement

Takeyoshi Tsutsui, Shinpei Kada, and Masaaki Saiki contributed to the diagnosis and treatment in this case report.

Takeyoshi Tsutsui, Masashi Oda, Shinpei Kada, and Masaaki Saiki contributed to Manuscript Development including writing the manuscript or providing critical revisions that are important for the intellectual content.

Takeyoshi Tsutsui and Kosuke Hayashi, Masashi Oda, Shinpei Kada, Naohiro Yamazoe, and Masaaki Saiki contributed to the approval of the final version of the manuscript files submitted to the Journal.

## Informed consent

Informed consent was acquired from the patient.

## Sources of funding

There was no source of funding in this case report.

## Ethical approval

The ethical approval was waived from our institution since all patients' identity was blinded.

## Registration of research studies

We have not yet registered this case report in “First in Man” case report.

## Guarantor

Corresponding Author (Takeyoshi Tsutsui) and Co-author (Masaaki Saiki) accept full responsibility.

## Declaration of competing interest

The authors report no conflicts of interest concerning the materials or methods used in this study or the findings specified in this paper.
